# Pathological Materia Medica

**Published:** 1882-11

**Authors:** Carroll Dunham


### PATHOLOGICAL MATERIA MEDICA.

The arrangement of materia medica on the basis of a pathologico-anatomical schema, as is desired by some, would be, first, impossible; second, useless; third, sure to mislead.—CARROLL DUNHAM.

